# Clinical significance of MSKCC nomogram on guiding the application of touch imprint cytology and frozen section in intraoperative assessment of breast sentinel lymph nodes

**DOI:** 10.18632/oncotarget.17490

**Published:** 2017-04-27

**Authors:** Lisha Sun, Guanglei Chen, Yizhen Zhou, Lei Zhang, Zining Jin, Weiguang Liu, Guangping Wu, Feng Jin, Kai Li, Bo Chen

**Affiliations:** ^1^ Department of Breast Surgery, The First Hospital of China Medical University, Shenyang, China; ^2^ Department of Surgical Oncology, The First Hospital of China Medical University, Shenyang, China; ^3^ Department of Breast Disease and Reconstruction Center, Breast Cancer Key Laboratory of Dalian, The Second Hospital of Dalian Medical University, Dalian, China; ^4^ Department of Pathology, The First Hospital of China Medical University, Shenyang, China

**Keywords:** early breast cancer, intraoperative detection, Memorial Sloan Kettering Cancer Center nomogram, touch imprint cytology, frozen section

## Abstract

The widely practiced intra-operative methods for rapid evaluation and detection of sentinel lymph node (SLN) status include frozen section (FS) and touch imprint cytology (TIC). This study optimized the use of TIC and FS in the intra-operative detection of breast SLNs based on the Memorial Sloan Kettering Cancer Center (MSKCC) nomogram. Three hundred forty-two SLNs were removed from 79 patients. SLN metastatic probability was assessed by the MSKCC nomogram. The SLNs underwent intra-operative TIC and FS, as well as routine post-operative paraffin sections (RPSs). The relationships between TIC, FS, and SLN metastatic probability were analyzed. Overall, TIC was more sensitive than FS (92.31% *vs*. 76.92%), while TIC specificity was inferior to FS specificity (84.85% *vs*. 100%). In addition, the best cut-off value for TIC based on the MSKCC nomogram was inferior to the best FS cut-off value (22.5% *vs*. 34.5%). All patients with a MSKCC value <22.5% in the present study were negative based on FS and RPS, while the true-negative and false-positive rates for TIC were 92.5% and 7.5%, respectively. Thus, early breast cancer patients, based on a MSKCC value <22.5%, can safely avoid FS, but should have TIC performed intra-operatively. Patients with a MSKCC value >22.5% should have TIC and FS to determine the size of metastases, whether or not to proceed with axillary lymph node dissection, and to avoid easily missed metastases.

## INTRODUCTION

As the most common malignant disease and the second leading cause of cancer mortality in women, breast carcinoma poses a threat to women's health [1, 2]. In recent years, sentinel lymph node (SLN) biopsies have become the standard for predicting overall axillary status in clinically node-negative breast cancer patients [3, 4]. Patients with pathologically-negative SLNs may safely avoid further axillary lymph node dissection (ALND), thus reducing complications, such as lymphedema, pain, upper limb movement disorders, and decreased quality of life [5–7]. Therefore, the intra-operative detection of SLNs has become a decisive factor in whether or not to proceed with ALND.

Currently, the most widely practiced intra-operative methods for rapid evaluation of SLN status include frozen section (FS) and touch imprint cytology (TIC) [4], while the majority of China's medical institutions consider FS for intra-operative diagnosis purposes. Numerous studies have shown that TIC has a sensitivity equivalent to or even better than FS. Moreover, TIC offers the advantages of minimal tissue preparation, good cytologic detail for interpretation, rapid staining with no loss of tissue, and no need for special equipment [8–13]. Therefore, how to effectively utilize TIC to reduce intra-operative misdiagnosis of SLNs and allow patients to safely avoid a second surgical procedure has become the focus of attention for surgeons.

Newly diagnosed breast cancer patients are often eager to learn more about their disease before undergoing a SLN biopsy. Memorial Sloan Kettering Cancer Center (MSKCC) [14] has developed a software program to allow easy calculation of the risk for metastasis based on nine variables prior to undergoing a SLN biopsy, including age, tumor size, histologic tumor type, lymphovascular invasion, tumor location, multi-focality, histologic grade, and estrogen and progesterone receptor status. With an area under the receiver operating characteristic (ROC) curve of 0.754, the nomogram is considered to be accurate and discriminating, and has been validated in a number of test groups [15, 16]. As a relatively comprehensive and scientific method to predict the metastatic risk for SLNs, the MSKCC nomogram has been increasingly used in clinical practice, but still cannot replace the SLN biopsy.

The primary aim of the present study was to compare TIC and FS for the intra-operative detection of SLNs in patients with early breast cancer. The secondary aim was to determine the clinical significance of the MSKCC nomogram with respect to the two methods for intra-operative assessment of breast SLNs.

## MATERIALS AND METHODS

### Patients and materials

A consecutive series of 79 patients with newly diagnosed invasive breast cancer who were treated in the Breast Surgery Department of the First Hospital of China Medical University between March 2014 and December 2014 were included. The inclusion criteria were as follows: (I) female gender; (II) 20-91 years of age; (III) breast tumor size range from 0.1-9.0 cm; (IV) axillary lymph nodes were clinically negative; and (V) the nine variables required for MSKCC nomogram prediction were available.

### Experimental materials and methods

We evaluated the SLN metastasis risk using the MSKCC nomogram through the internet for each newly diagnosed breast cancer patient. The SLNs were freshly dissected along the longitudinal axis at 2.0-mm intervals after excluding the adipose envelope, and each cut surface was used for touch imprinting at least twice onto a clean glass slide. When the slide was semi-dried, the slide was immersed in 95% ethanol solution for 3 min, followed by hematoxylin and eosin (H&E) staining.

After touch imprinting, all SLNs were processed for intra-operative FSs as well as post-operative routine paraffin sections (RPSs), which is the standard protocol in our hospital. RPSs served as the gold standard for evaluating intra-operative techniques. TIC results were blinded to pathologists performing FSs. The results of FSs were used by the surgeon to determine whether or not ALND should be performed, but no decision based on the TIC result was made.

The pathologic results were classified as macrometastases (>2.0 mm), micrometastases (0.2-2.0 mm), and isolated tumor cells (ITCs, <0.2 mm) according to the TMN staging system [17]. Patients with intra-operatively positive FS (except for ITCs) required an immediate ALND with or without an instant prosthesis, while patients with intra-operatively negative FSs, but post-operatively positive RPSs (except for ITCs) proceeded with a second ALND.

### Statistical analysis

The results of TIC and FS were compared with the post-operative RPSs and were classified as true-positive (TP), true-negative (TN), false-negative (FN), or false-positive (FP), on a patient basis. True-positive cases were cases that were shown to contain carcinomas, both on intra-operative pathology and post-operative RPSs. The formulas used to calculate statistical parameters were as follows: sensitivity = TP/ (TP + FN); specificity = TN/ (TN + FP); overall accuracy = (TP + TN)/ (TP + FP + TN + FN); negative predictive value (NPV) = TN/ (TN + FN); and positive predictive value (PPV) = TP/ (TP + FP).

All data were analyzed with SPSS statistical software (version 17.0; SPSS, Inc., Chicago, IL, USA). Differences in sensitivity and accuracy between the two intra-operative pathologic methods were determined using paired chi-square or Fisher's exact tests, and the best cut-off value was established based on ROC curve analysis. A *P*-value < 0.05 was considered statistically significant.

## RESULTS

### Patient and tumor characteristics

Seventy-nine newly diagnosed breast cancer patients were enrolled in this study. The median age of the patients was 50 years (range, 26-76 years). The primary tumor size ranged from 0.4-3.5 cm (median, 1.3 cm; interquartile range, 0.8-1.7 cm). The tumor pathologic types were as follows: invasive ductal carcinoma, 74 (93.7%); invasive lobular carcinoma, 2 (2.5%); and special type of carcinoma, 3 (3.8%). Of the patients, 24.1% had primary tumors located within the upper inner quadrant of the breast and 6.3% were confirmed to have multi-focal tumors. None of the patients had lymphatic or vascular invasion. The hormone receptor status of primary tumors was as follows, as shown in Table 1: ER-positive (ER≥10%), 61; PR-positive (PR≥10%), 51.

**Table 1:** Patient and tumor characteristics based on MSKCC nomogram

Number of patients	79
Age ,years Median Range	50 26-76
Primary tumor size, cm Median IQR*	1.3 0.8-1.7
Primary tumor histological type	
Ductal (%)	74 (93.7)
Lobular (%)	2 (2.5)
Special type (%)	3 (3.8)
Lymphovascular invasion	0
Upper inner quadrant (%)	19 (24.1)
Multifocality (%)	5 (6.3)
Histological grade	
Ductal I (%)	24 (30.4)
Ductal II (%)	52 (65.8)
Ductal III (%)	1 (1.3)
Lubular (%)	2 (2.5)
Primory tumor hormone receptor status	
ER≥10% (%)	61 (77.2)
PR≥10% (%)	51 (64.6)

*IQR= Inter Quartile Range

### Pathologic results of SLN biopsies

**Figure 1:**
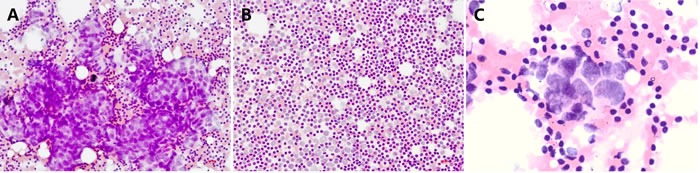
Representative photomicrographs of TIC staining of SLNs **A.** A ‘true-positive’ sentinel lymph node that was positive with both intra-operative TIC and post-operative RPS (H&E; magnification, ×200); **B.** A ‘true-negative’ sentinel lymph node that was negative with both intra-operative TIC and post-operative RPS (H&E; magnification, ×200); **C.** A ‘false-positive’ sentinel lymph node that was positive with TIC, but negative with RPS (H&E; magnification, ×400).

In the current study, all 79 patients underwent SLN biopsies. There was a total of 342 SLNs (an average of 4.3 SLNs per patient). Two hundred ninety-nine SLNs were dissected along the longitudinal axis and had touch imprints. All SLNs were processed for intra-operative FSs, as well as post-operative RPSs, which is the standard protocol in our hospital. Based on the post-operative RPSs, among the 79 cases, 13 (16.5%) had at least 1 positive SLN. The other pathologic results were as follows: TIC-positive, 22; and FS-positive, 10. There were 56 cases of TIC(-)FS(-)RPS(-), 10 cases of TIC(+)FS(+)RPS(+), 2 cases of TIC(+)FS(-)RPS(+), 1 case of TIC(-)FS(-)RPS(+); and 10 cases of TIC(+)FS(-)RPS(-). Figure 1A-1C shows the H&E staining images of SLN TICs.

With post-operative RPSs as the histopathologic diagnostic criteria, the sensitivity, specificity, and accuracy of TIC and FS were 92.31%, 84.85%, and 86.08% versus 76.92%, 100%, and 96.20%, respectively (Table 2). Both methods had great diagnostic accuracy because of the approximately equal Youden index (0.772 vs. 0.769), but the sensitivity difference of the two methods was statistically significant (*P*<0.01). In addition, the NPV and PPV of TIC and FS were 98.25% and 54.55% versus 95.65% and 100%, respectively (Table 2).

**Table 2:** Pathologic results of TIC and FS by case, respectively (*n* = 79)

Method	TP^#^	FN^#^	TN^#^	FP^#^	Sensitivity	Specificity	Accuracy	NPV^#^	PPV^#^
TIC	12	1	56	10	92.31%	84.85%	86.08%	98.25%	54.55%
FS	10	3	66	0	76.92%	100.00%	96.20%	95.65%	100.00%

^#^ TP, True Positive; FN, False Negative; TN, True Negative; FP, False Positive; NPV, negative predictive value; PPV, positive predictive value.

### The relationship of TIC, FS, and SLN metastatic risk predicted by the MSKCC nomogram

**Figure 2:**
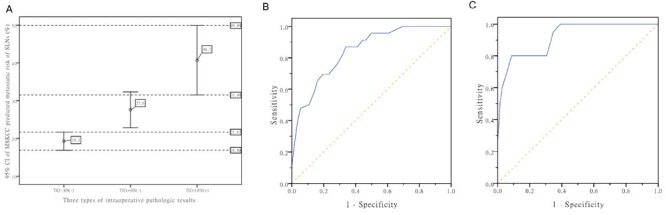
The relationship of TIC, FS, and SLNs metastatic risk predicted by the MSKCC nomogram **A.** MSKCC predicted SLN metastatic risk ranges of TIC(-)FS(-), TIC(+)FS(-), and TIC(+)FS(+) [95% CI]. **B.** The ROC curve of MSKCC prediction for TIC. **C.** The ROC curve of MSKCC prediction for FS.

The metastatic risk probability of SLNs was calculated by the MSKCC nomogram for all enrolled patients. In the present study, the SLN metastatic risk ranges for TIC(-)FS(-), TIC(+)FS(-), and TIC(+)FS(+) as assessed by the MSKCC nomogram were 4%~39%, 14%~38%m and 25~63%, respectively (95% confidence interval: 16.88~21.67%, 22.86~32.37%, and 31.49~49.89%, respectively), and the best cut-off value based on the MSKCC prediction for TIC was inferior to FS (22.5% and 34.5%, respectively). These results are shown in Figure 2A-2C.

According to Figure 2B and 2C, the ROC curve for TIC is closer to a smooth curve, which indicates that the cut-off value of 22.5% is statistically convincing. A large polyline appears in the ROC curve for FS, indicating that the cut-off value of 34.5% is not statistically accurate. It may be possible to avoid this situation with more cases; however, based on the current data, the cut-off value of TIC is more convincing than FS. Thus, we only calculated the rate of TP, TN, FP, and FN based on the cut-off value of 22.5%. All patients with a MSKCC value <22.5% in this study were FS- and RPS-negative, while the TN and FP rates for TIC were 92.5% (37/40) and 7.5% (3/40), respectively. For patients with a MSKCC value >22.5%, the TP, TN, FP, and FN rates for FS were 25.64% (10/39), 66.67% (26/39), 0, and 7.69% (3/39), respectively. The corresponding rates for TIC were 30.77% (12/39), 48.72% (19/39), 17.95% (7/39), and 2.56% (1/39), respectively. Detailed data are shown in Table 3.

**Table 3:** The rate of TP, TN, FP and FN based on the cutoff value of 22.5%

	<22.5% (*n*= 40)		<22.5% (*n*= 40)	
	FS	TIC	FS	TIC
TP	0	0	25.64% (10/39)	30.77% (12/39)
TN	100%(40/40)	92.5% (37/40)	66.67% (26/39)	48.72% (19/39)
FP	0	7.5% (3/40)	0	17.95% (7/39)
FN	0	0	7.69% (3/39)	2.56% (1/39)

## DISCUSSION

Over the past decade, many researchers are keen to apply molecular assays to improve intra-operative pathologic accuracy of breast SLNs, such as the Metasin assay [18, 19] and one-step nucleic acid amplification assay (OSNA) [20, 21], but neglect to give full consideration to the existing mature pathologic techniques (TIC and FS), which are widely used in clinical practice. In addition, a study of OSNA in 552 patients showed both OSNA and TIC can serve as qualified intra-operative assessments of SLNs, and suggested that OSNA can be applied as a complement to histopathologic assessment, but cannot replace pathology with serial sectioning [22].

The main concern about FS for surgeons is that the procedure may result in the loss of lymphoid tissue, thus affecting the accuracy of intra-operative assessment of SLNs, and even affecting the results of post-operative pathologic diagnosis [23, 24]. Using RPS as the gold standard, there were no cases that were FS-positive, but RPS-negative in the present study. Three FS-negative cases were detected with micrometastases by RPS post-operatively. TIC is a technique that is often overlooked, but has a complementary effect on tissue sections. TIC is convenient, timesaving, low cost, and the procedure is relatively simple with no loss of specimen; however, TIC is prone to atypical results because of fewer cells and no complete organizational structure [25–27]. According to our results, there were 10 cases of TIC considered to be FP when using RPS as the gold standard. As shown in Figure 1C, malignant cells were observed in these 10 cases that were considered to be positive by the cytopathologists. The high rate of TIC-positive, but RPS-negative cases in our findings suggests that TIC may detect some metastastic SLNs that are easily missed by RPS, which is consistent to the results of Motomura [13]. In contrast, improving the procedure for pathologic examinations, or taking other more exhaustive techniques as gold standards, such as serial paraffin sections [28, 29] and OSNA [30, 31], the FP rate for TIC may be decreased, while the FN rate may be simultaneously increased.

The performance of TIC varies significantly among institutions [22]. The results of the current study showed the sensitivity of TIC was significantly better than FS (92.31% vs. 76.92%, *P*<0.01), which is in agreement with previously published data [13, 32], but inconsistent with other data [33]. We considered the reason for this finding be the sampling bias of SLNs when preparing for TIC and FS. Specifically, TIC can avoid the tissue loss that inevitably occurs in the cryostat. The range of capabilities of cytopathologists may be another reason because sinus histiocytosis with large vesicular nuclei and distinct nucleoli may be falsely labeled as malignant on TIC [11].

Related studies involving SLNs have indicated that low sensitivity and a high FN rate for TIC is mostly caused by micrometastases in the study sample [34]. Micrometastases can be detected only after exhaustive examination with serial sectioning and CK19 immunostaining [29]. Nevertheless, some researchers have reported that TIC may detect more micrometastases in SLNs [13, 28]. There was only one case of a FN TIC in the current study, as shown in Table 2, so we did not calculate the FN rate occurred because of ITC or micrometastases.

Metastatic lobular carcinoma is difficult to identify in SLNs because of the low-grade cytomorphology, the tendency to infiltrate lymph nodes in a single cell pattern, and because individual cells can resemble lymphocytes [9]. Howard et al. [35] found that the sensitivity, specificity, and accuracy of pure invasive lobular cancer were 71%, 100%, and 92%, respectively. No statistically significant differences were identified between the intra-operative detection of lobular carcinoma versus ductal carcinoma [35]. Moreover, Wang et al. [36] found that TIC can be used as a reliable method for detecting SLN metastasis only in young patients with invasive lobular carcinoma. The number of cases of invasive lobular carcinoma in this study was small and there were no FN results in these cases, thus we have not calculated the FN rate in this group of patients.

In the present study, 69 of 79 patients had FS-negative SLNs on biopsy, and 10 patients received instant prostheses. One of the patients who received an instant prosthesis was RPS-positive post-operatively as well as TIC-positive according to our data, thus it was inappropriate for this patient to receive an instant prosthesis. Therefore, the combination of TIC and FS to improve the intra-operative pathologic accuracy of SLNs is of great clinical significance. Furthermore, a number of comparative studies involving TIC and FS are in agreement that the combination of TIC and FS can improve any one diagnostic method [33, 37].

While blindly performing TIC and FS for each patient will increase the workload of surgeons, how can we effectively take advantage of TIC and FS from SLN biopsies? The positive predictive value of FS was significantly superior to TIC (100% vs. 54.55%), while the negative predictive value of TIC was superior to FS (98.25% vs. 95.65%), suggesting that FS-positive results were more consistent with RPSs, and TIC was more suitable to exclude negative pathologic results. Moreover, the best cut-off value for MSKCC prediction based on TIC was 22.5%. All patients with a MSKCC value <22.5% in the current study had negative FS and RPS, but the TN and FP rates for TIC were 92.5% (37/40) and 7.5% (3/40), respectively (Table 3), indicating that early breast cancer patients with a MSKCC value < 22.5% can safely avoid FS, but only receive TIC intra-operatively, thus saving operative time, and more importantly, avoiding frozen damage caused by FS and leaving more comprehensive tissue for post-operative pathologic examination. For intra-operative TIC-positive cases, it is recommended to make the decision whether or not to perform ALND until the post-operative pathologic results are available, thus avoiding unnecessary ALND.

For patients with a MSKCC value > 22.5%, because of the higher probability of SLN metastases, it is suggested that intra-operative TIC should be combined with FS to measure the size of metastases, and determine whether or not to proceed with ALND, and to avoid easily missed metastases. For intra-operative TIC-positive, but FS-negative cases, it is recommended to make the decision whether or not to perform ALND until post-operative pathologic results are determined.

In conclusion, we believe that our findings provide innovative insight into the MSKCC nomogram and intra-operative assessment of breast SLNs. We determined the best cut-off value for MSKCC prediction based on TIC, and appropriate intra-operative techniques to assess the status of SLNs, thus saving operative time for some patients and leaving more tissue for post-operative pathologic examination. Most importantly, the accuracy of intra-operative assessment of SLNs can be improved and easily missed metastases can be avoided. Due to the small size of enrolled patients in the current study, it is necessary to confirm with a larger amount of data when to apply the results in clinical practice.
